# The efficacy of gaseous ozone against different forms of *Candida albicans*

**DOI:** 10.29252/cmm.3.2.26

**Published:** 2017-06

**Authors:** M Zargaran, M Fatahinia, A Zarei Mahmoudabadi

**Affiliations:** 1 **Infectious and Tropical Diseases Research Center, Health Research Institute, Ahvaz Jundishapur University of Medical Sciences, Ahvaz, Iran**; 2 Department of Medical Mycology, School of Medicine, Ahvaz Jundishapur University of Medical Sciences, Ahvaz, Iran

**Keywords:** Amphotericin B, *Candida albicans*, Caspofungin, Fluconazole, Gaseous ozone

## Abstract

**Background and Purpose:**

Ozone is an inorganic molecule with effective antimicrobial properties. Clinical treatment of ozonated water was used for the elimination of *Candida albicans*, *Enterococcus faecalis*, endotoxins, and biofilms from root canals. In addition, its therapeutic effects for tinea pedis, ulcers, and leishmaniasis were investigated. The purpose of the present study was to evaluate the fungicidal effects of ozone on different forms of* C. albicans*. In addition, antifungal susceptibility profile of strains was assessed before and after exposure to ozone.

**Materials and Methods:**

Fifty strains of *C. albicans* were exposed to gaseous ozone at different times. Furthermore, biofilm formation and germ tube production were evaluated when yeast suspensions were exposed to ozone. In addition, antifungal susceptibility of ozone resistant colonies was investiagted as compared to controls.

**Results:**

Ozone was highly effective in killing *C.* *albicans *in yeast form and inhibition of germ tube formation during 210 and 180 s, respectively. Although with increasing exposure time biofilm production was considerably decreased, resistance to ozone was much higher among vaginal and nail isolates even after 60 min. All the strains were sensitive to fluconazole, caspofungin, and terbinafine pre- and post-ozone exposure. Resistance to amphotericin B was significantly enhanced after exposure to ozone.

**Conclusion:**

Although ozone was highly effective on the yeast form of *C.* *albicans* and it can inhibit the formation of germ tubes in *C.* *albicans*, the complete removal of biofilms did not happen even after 60 min. It seems that ozone therapy induces resistance to amphotericin B.

## Introduction

Ozone, trioxygen (O_3_), is a highly reactive inorganic molecule. It is a natural part of the atmosphere also produced by ozone generators. Although ozone was discovered in the mid-nineteenth century, its medicinal usage was discovered during the recent decades. It was found that ozone is one of the best microorganism killers. As such, it has antibacterial, antiviral, antifungal, and anti-parasitic properties [1-3]. In addition, ozonated oils (oleozone) are used in the medical and pharmaceutical products [4] and in textile [3] and food industries [5]. 

Decontamination of medical wastes in hospitals is another application of ozone that was first proposed by Coronel et al. [6]. Furthermore, in a study, ozonated water reduced the CFU/ml of *Candida albicans* on denture plates [7]. Some other applications of ozone are disinfection and removing the toothbrushes bristles microbiota [8] and as a fungicidal and detoxifying agent of aflatoxins in peanuts [9]. Some studies have shown that the combination of ozone with chlorhexidine 2% had synergistic effects against *C. albicans* and *Enterococcus faecalis* [10]. Moreover, ozonized olive oil showed antifungal activity against dermatophytes such as *Microsporum canis* and *Trichophyton rubrum* [11].

Recently, clinical treatments by ozonated water have been used for the elimination of *C. albicans* [2], *E. faecalis* [2], endotoxins [2], and biofilms [12] from root canals. In addition, ozonated water is applied for the sterilization of contaminated dental instruments with *Escherichia coli*, *Staphylococcus aureus*, *C.* *albicans,* and *Bacillus* *atrophaeus* spores [13]. Therapeutic effects of ozone on arthritis [4], cutaneous infections [1], tinea pedis [14], and ulcers [4] were investigated by different researchers. Zanardi et al. evaluated the effects of ozonated oils for the treatment of cutaneous infections; they concluded that these compounds are highly promising in the treatment of cutaneous infections [1]. Furthermore, in a study on recurrent vulvovaginal candidiasis, it was found that ozonized distilled water cured the disease among 85% of patients, 10% remained asymptomatic, and only 5% did not respond to ozone therapy [15]. The efficacy of ozonated olive oil against *Leishmania* promastigote was investigated by Rajabi et al., who noted that its effect was dose dependent [16].

***Objectives***

The purpose of the present study was to evaluate the fungicidal effects of gaseous ozone on some strains of *C. albicans* in yeast form, germ tube formation, and biofilm production. In addition, antifungals susceptibility profile of the tested strains was assessed before and after ozone exposure.

## Materials and Methods

***Candida albicans*** **strains**

Fifty *C. albicans* strains originating from different sources were used for the in vitro experiments. Ten isolates from patients with vaginitis, 10 isolates from cases of nail infection, 10 isolates from oral cavity of neutropenic patients, and 10 isolates from urine samples of patients with candiduria were clinically isolates. All the *C. albicans *strains were previously identified and stored in the Department of Medical Mycology, Ahvaz Jundishapur University of Medical Sciences, Ahvaz, Iran. All the 40 strains were re-identified using the routine mycological tests including morphology and microscopy on Corn Meal Agar (Difco, USA) with 1% Tween 80, germ tube formation, growth at 45ºC, and green colony production on CHROMagar *Candida *(CHROMagar *Candida*, France) [17]. Furthermore, 10 environmental strains of *C. albicans* were isolated from environmental materials and detected using the above-mentioned tests. 

***The influence of ozone on the inhibition of Candida albicans growth***

An overnight culture of each strain was prepared on Sabouraud Dextrose Agar (SDA) plates (Biolife, Italy) and incubated at 37ºC. Suspensions were prepared in sterile distilled water and standardized using 0.5 McFarland (1-5×10^6 ^CFU/ml). In the present study, gaseous ozone was freshly produced by an ozone gas generator machine (Purus WA600, Canada). The concentration of the produced ozone was 400 mg/h. Eight sterile falcon tubes containing 5 ml of diluted standard yeast suspension (1:2) was prepared (0.5-2.5×10^6^ CFU/ml). Seven suspensions were exposed to gaseous ozone for 30, 60, 90, 120, 150, 180, and 210 s using flow of ozone gas by a sterile micropipette tip. The eighth suspension falcon was selected as the positive control. Then, 5 µl of each suspension was inoculated on SDA plates and incubated at 37ºC for 48 h. Growth colonies were counted and CFU/ml was calculated [18]. 

***The influence of ozone on the inhibition of germ tube formation of Candida albicans***

An overnight culture of the tested *C. albicans* strains on SDA was prepared, and then a loopful of a colony was suspended in 0.5 ml of fresh human serum. For each strain, seven tubes containing 0.5 ml of yeast suspension were prepared. Six tubes were exposed to gaseous ozone for 30, 60, 90, 120, 150, and 180 s and one tube was used as control without ozone exposure (as described above). The tubes were incubated at 37ºC for 3 h, and then they were examined microscopically for germ tube formation. The presence of germ tubes was counted and compared with control (3+) as 3+, 2+, 1+, and negative. 

***The influence of ozone on biofilm formation of Candida albicans***

Biofilm formation was performed as previously described [19]. Briefly, 20 µl of each yeast suspension and 180 µl of SDA supplemented with 8% glucose (Merck, Germany) were added to each microplate well. Seven series of inoculated microplates were prepared. Microplates were incubated at 37ºC for 48 h and then washed with sterile phosphate-buffered saline (PBS) in trice. Each microplate was separately put in a sterile zipped plastic bag and exposed to a flow of ozone for 15, 20, 30, 40, 50, and 60 min. One microplate was selected as positive control. Then, 200 µl of RPMI-1640 (Bio IDEA, Iran) that contained 0.01% resazurin (Sigma-Aldrich, Germany) was added to each well and incubated at 37ºC for 24-48 h. Viable biofilm cells can grow and change the blue color to red.

***The influence of ozone on antifungal susceptibility testing***

A stock solution of 32 mg/ml for amphotericin B (Sigma-Aldrich, Germany), fluconazole (Serva, USA), and terbinafine (Sigma-Aldrich, Germany) and1.25 mg/ml of caspofungin (Sigma-Aldrich, Germany) were prepared in dimethyl sulfoxide (DMSO, Fluka, Germany) and stored at -20ºC. Furthermore, a serial dilution of each antifungal was prepared with RPMI-1640 0.01% resazurin, including amphotericin B from 0.125 to 16 µg/ml, fluconazole from 0.125 to 16 µg/ml, terbinafine from 2 to 256 µg/ml, and caspofungin from 0.03125 to 4 µg/ml.

A standard suspension of overnight growth colonies of each strain was prepared before and after ozone exposure. The suspensions were diluted using RPMI-1640 0.01% resazurin [20]. Then, 100 µl of diluted suspensions and 100 µl of serial dilution of antifungals were inoculated into each microplate well [21, 22]. The microplates were incubated at 37ºC for 24-48 h, and then minimum inhibitory concentration (MIC) for each strain was detected based on indicator changes. 

***Statistical analysis***

The obtained data were calculated as frequency and presented in the four figures and one table. In addition, the sensitivity of isolates to amphotericin B and terbinafine was analysed by using Chi-squared test in SPSS, version 22. *P-value* less than 0.05 was considered statistically significant. 

## Results

**Figure 1:**
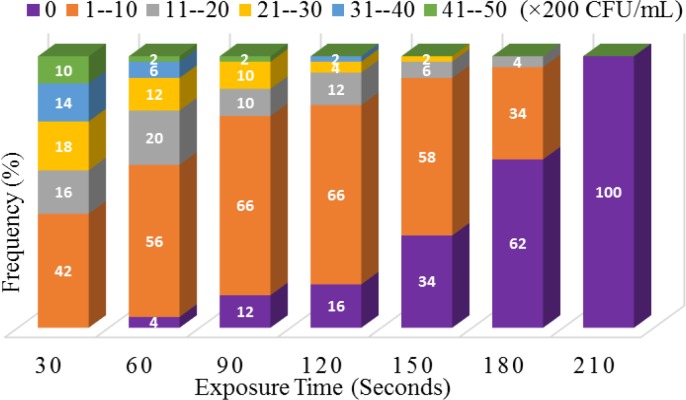
The effect of ozone on colony forming units of Candida albicans

Our results revealed that ozone is highly effective in killing *C.* *albicans* since a reduction of the microorganism concentration was observed during the contact time with ozone. This reduction was initially started after 30 s of zone exposure and fully observed after 210 s (Figure 1).

**Figure 2:**
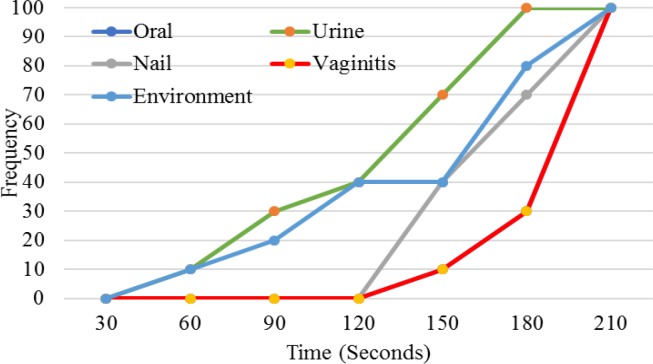
The effect of ozone on Candida albicans from different sources

As shown in Figure 2, vaginal and oral isolates were more resistant to ozone followed by nail, environmental, and urine isolates. Our study demonstrated that only urine isolates were fully inactivated at 150 s, whereas the other isolates were completely inhibited after 210 s. 

**Figure 3:**
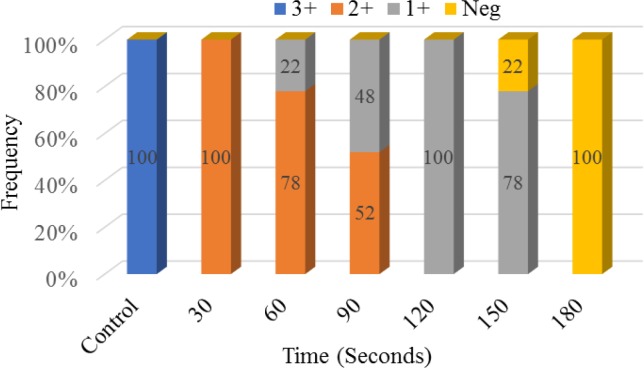
The effect of ozone on germ tube formation in Candida albicans

In the present study, we examined the effect of ozone on germ tube production in *C. albicans* strains at different times. Our results presented that the production of germ tube in *C. albicans* could be affected by different exposure times to ozone. In comparison with control (germ tube formation=3+), with increasing exposure time, the total number of germ tubes decreased (30 s). Furthermore, after 150 s exposure to ozone, 24% of the isolates failed to form germ tubes, and germ tube formation was completely inhibited after 180 s (Figure 3).

**Figure 4:**
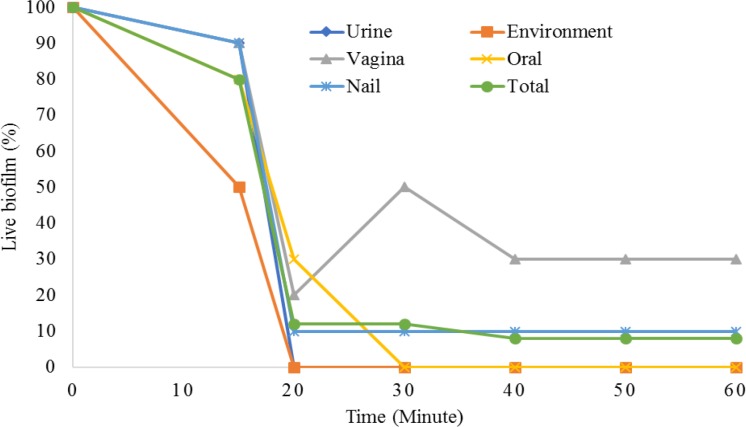
The effect of ozone on biofilm formation in Candida albicans

The sensitivity of *C. albicans* biofilm against ozone was also evaluated. Generally, as shown in Figure 4, with increasing exposure time, biofilm formation was considerably diminished. However, resistance to ozone was much higher among vaginal isolates, followed by nail strains. Other strains were completely inhibited at 20 or 30 min.

***The impact of ozone on susceptibility of Candida albicans to antifungals***

The tested *C. albicans* strains were susceptible to fluconazole with MIC ranging from < 0.125 to 4 µg/ml before ozone exposure, whereas this range decreased to <0.125-2 µg/ml after exposure (Table 1). MIC_50_ and MIC_90_ of fluconazole against the isolates were respectively 0.5 and 1 for both conditions, pre- and post-ozone therapy. In general, all the isolates were sensitive (MIC ≤ 8 *μg*/*ml*) to fluconazole in both conditions. The details of sensitivity of the isolates to caspofungin are presented in Table 1. The effect of caspofungin against the strains was completely similar pre- and post-ozone exposure, that is, all the strains were within the MIC range of <0.031-1 µg/ml. In addition, MIC_50_ and MIC_90_ were 0.25 and 0.5 for both conditions, respectively. All the isolates pertainedto the sensitive range (susceptible, MIC < 1) of caspofungin.

Although the MIC range of terbinafine against the isolates was similar before and after exposure to ozone (<2 - >256 µg/ml), its MIC_50_ was considerably different (<2 µg/ml before and 4 µg/ml after exposure). However, there was no significant difference (*P=0.689*) among the isolates in susceptibility to terbinafine. When the strains were exposed to ozone, their MIC range and MIC_90_ for amphotericin B were completely similar to the unexposed ones (<0.125-16 and 8 µg/ml). On the other hand, MIC_50_ was increased from <0.125 to 1 after ozone exposure (Table 1). Overall, there was a significant difference (*P=0.006*) in the effect of ozone on the sensitivity of the isolates to amphotericin B.

MIC breakpoints set by Clinical and Laboratory Standards Institute were as follows: for amphotericin B, (susceptible, MIC < 2 *μg*/*ml*; resistant *MIC ≥2 μg*/*ml*) [23], terbinafine (susceptible, MIC ≤8 *μg*/*ml*; resistant, MIC >8 µg/ml) [24], fluconazole (susceptible, MIC ≤ 8 *μg*/*ml*; dose dependent, 16-32* μg*/*ml;* resistant, MIC ≥ 64 µg/ml) [25], caspofungin (susceptible, MIC < 1 *μg*/*ml*; resistant, MIC ≥ 2µg/ml) [26].

**Table 1:** MIC range, MIC_50_, and MIC_90_ for the isolates against antifungal agents before and after exposure to ozone

**Antifungals**		**Isolates sources**										**Total**		***P Value***
		**Vaginitis**		**Oral cavity**		**Nail**		**Urine**		**Environment**				
		**Before**	**After**	**Before**	**After**	**Before**	**After**	**Before**	**After**	**Before**	**After**	**Before**	**After**	
Fluconazole	MIC range	0.5-4	0.5-1	<0.125-1	<0.125-1	0.25-1	0.25-1	0.5-2	0.5-2	0.5-1	0.5-2	<0.125-4	<0.125-2	No resistant observed
	MIC_50_	0.5	1	<0.125	<0.125	0.25	0.25	0.5	0.5	0.5	0.5	0.5	0.5	
	MIC_90_	2	1	<0.125	<0.5	0.5	0.5	1	1	1	1	1	1	
Caspofungin	MIC range	0.125-0.5	0.25-0.5	<0.031-0.25	<0.031-0.25	0.0625-0.5	0.0625-0.25	0.25-0.5	0.25-0.5	0.25-1	0.25-1	<0.031-1	<0.031-1	No resistant observed
	MIC_50_	0.25	0.25	0.125	0.25	0.25	0.25	0.5	0.5	0.25	0.5	0.25	0.25	
	MIC_90_	0.5	0.5	0.25	0.25	0.25	0.25	0.5	0.5	1	1	0.5	0.5	
Amphotericin B	MIC range	<0.125-8	<0.125-4	<0.125	<0.125-4	<0.125-16	<0.125-8	<0.125	<0.125-16	<0.125-16	<0.125-8	<0.125-16	<0.125-16	P=0.006
	MIC_50_	<0.125	<0.125	<0.125	<0.125	8	1	<0.125	4	<0.125	1	<0.125	1	
	MIC_90_	<0.125	8	<0.125	4	16	8	<0.125	8	<0.125	4	8	8	
Terbinafine	MIC range	<2->256	<2->256	<2->256	<2->256	<2->256	<2->256	<2->256	<2-256	<2-256	<2->256	<2->256	<2->256	P=0.689
	MIC_50_	256	256	<2	<2	256	256	<2	<2	<2	<2	<2	4	
	MIC_90_	>256	>256	>256	>256	256	>256	256	16	256	>256	>256	>256	

## Discussion

Ozone is a chemical compound composed of three oxygen atoms that has higher energy content than atmospheric oxygen. This compound has medicinal (for disinfecting and treating microbial infections and non-microbial diseases), industrial, and agricultural applications [1, 2, 4, 7, 9, 27]. Ozone is used in the treatment of recurrent vulvovaginal candidiasis [15], and it has synergistic effect with chlorhexidine for the treatment of infected root canals [10].

In the present study, an ozone generator with an output of 6.7 mg/min of ozone started inactivation of the yeasts at 30 s, and fungicidal activity was fully completed after 210 s. In a study by Restaino et al., the inactivation of a 10^4^-10^6^ CFU/ml suspension of *C. albicans *was observed after 5 min of ozone exposure [5]. da Silva Faria et al. believed that clinical strains of *Candida* were more resistant to ozone than non-clinical isolates. They found that ozone exposure for 5 min was not fully effective in the inactivation of these types of isolates [18]. Furthermore, in a studyby Cardoso et al., the complete inactivation of *C. albicans* was observed after 40 min of exposure to ozonated water with 600 ppm ozone [2]. On the other hand, in brushes bristles exposed to ozonated PBS, complete sanitation was observed after 30 min [8]. Our results indicated that the most resistant isolates to ozone were those of the oral cavity and vulvovaginal candidiasis (same effect). Whereas urine isolates of *C. albicans* were more sensitive to ozone than the other isolates, such that after 150 s of exposure, they were fully inhibited by ozone. It seems that other factors, such as the initial suspension concentration and sources of isolates, could affect fungicidal activity of ozone. 

Germ tube formation in *C. albicans* is the first step in adhesion to host tissues and acrylic surfaces, therefore, it is considered as one of the important pathogenic factors. In a study by Ali et al. on diabetic foot ulcer isolates, germ tube formation in *C. albicans* was completely inhibited when exposed to 3 ppm for 180 min [28]. In contrast, in our study, germ tube inhibition started at 60 s exposure and completely stopped at 180 s. 

The biofilms of *C.* *albicans* are resistant to several antifungals and their elimination plays a major role in the treatment of the disease [29]. The previous studies have shown that ozone is a suitable tool for the removal of biofilm from dentures [30]. Therefore, considering the formation of biofilms, especially in catheters, urethral catheters, and dentures, and their resistance to antifungals, it can be stated that ozone gas can help eliminate biofilms. Interestingly, in our study, biofilms produced by vaginal and nail isolates showed greater resistance to ozone than the other isolates, which is in accordance with its yeast form resistance. In a study, 30 min exposure of acrylic dentures to high concentrations of ozonated water at 60°C completely removed *Candida* biofilms [31]. In another study on yeast biofilm, it was found that the disinfecting action of ozone was less effective than the yeast form [32]. In our study, vaginal and nail isolates were resistant to ozone even after 60 min of exposure, which was confirmed by Müller et al., who believed that ozone was not able to completely eliminate biofilms [33]. 

Geweely has shown that ozonized olive oil (oleozone) damages DNA and RNA in *C. albicans* [11]. Furthermore, effect on microorganism extracellular enzymes, as well as oxidation of phospholipids and lipoproteins are the other effects reported by researchers [4]. There are few reports on DNA damages caused by ozone and their association with antibiotic sensitivity. In addition, data regarding susceptibility to antifungal drugs in *Candida* species exposed to ozone is not available. Therefore, if ozone-resistant isolates are modified and resistant to antifungal drugs, they can aggravate the risk of disease and failure to respond to antifungal drugs. Our study did not reflect any changes in the sensitivity of *C. albicans* to fluconazole and caspofungin after exposure to ozone. All the isolates were sensitive to both antifungal agents before and after exposure to ozone. Although the number of resistant isolates to terbinafine increased from 23 to 25, this difference was not significant (*P=0.689*).In contrast, resistance to amphotericin B among *C. albicans* isolates increased significantly after ozone exposure (*P=0.006*). Thus, it seems that ozone induces resistance to amphotericin B, while it does not change sensitivity or resistance to other antifungal agents.

## Conclusion

Ozone is particularly effective in the growth of the yeast forms of *C.* *albicans* and has high fungicidal activity at 210 s exposure. Furthermore, it can prevent the formation of germ tubes in *C.* *albicans*; however, the complete removal of biofilms was not possible even after 60 min. It seems that ozone therapy was not effective in inducing resistance to fluconazole, caspofungin, and terbinafine in *Candida* isolates, with the exception of amphotericin B, against which resistance was significantly enhanced after ozone exposure.
